# Comparing Intradermal (ID) Rabies Vaccination with Conventional IM Regimen on Humoral Response of New Zealand White Rabbits for the Production of Animal-Derived Polyclonal Antibodies

**DOI:** 10.1155/2024/4451881

**Published:** 2024-05-18

**Authors:** Amina Najam, Rameesha Abid, Hussain Ali, Hamza Hafeez, Amna Arif, Safia Ahmed, Alessandro Di Cerbo, Shakira Ghazanfar

**Affiliations:** ^1^Biological Production Division, National Institute of Health, Islamabad 45500, Pakistan; ^2^Department of Microbiology, Quaid-I-Azam University, Islamabad 44100, Pakistan; ^3^National Institute for Genomics and Advanced Biotechnology (NIGAB), National Agricultural Research Centre, Park Road, Islamabad 45500, Pakistan; ^4^Department of Biological Sciences, University of Sialkot, Sialkot 51310, Pakistan; ^5^Department of Applied Microbiology, University of Veterinary and Animal Sciences, Lahore 05499, Pakistan; ^6^School of Biosciences and Veterinary Medicine, University of Camerino, Matelica 62024, Italy

## Abstract

In developing countries, it is imperative to implement cost-effective strategies for animal humoral response development in the production of antiserum. This study compared the effect of immunization regimens on the humoral immune response of New Zealand White (NZW) rabbits (*N* = 24) using cell culture rabies vaccine (CCRV) through intradermal (ID) and traditional intramuscular (IM) routes. The rabbits were divided into three experimental groups: (a) IPC-R2 with a two-site one-week regimen; (b) TRC-R3 with a two-site twenty-eight-day regimen; and (c) Alternate-R4 with a four-site one-week regimen. These regimens were then compared to the standard IM schedule of five doses of rabies vaccine administered at days 0, 3, 7, 14, and 28 in control group R-1. The results were evaluated at days 14 and 35 postvaccination using rabies-specific Platelia II™ ELISA kit method. The results showed a better response to the ID regimen than the IM route regarding immunogenicity and volume consumption of the vaccine. The three selected ID regimes showed significantly higher mean titer values than the control IM regimen group R-1 (*p* < 0.001). The study aims to explore simple immunization strategies to enhance the RV-specific antibody titers for immunization donor animals. This method would produce polyclonal antibodies and strengthen local production of polyclonal antibodies in Pakistan to deal with vaccine and rabies immunoglobulin (RIG) shortage, thus providing effective postexposure prophylaxis (PEP) for better control of rabies in developing countries.

## 1. Introduction

Rabies is a zoonotic viral disease that is fatal to animals and humans. According to recent statistics, 59,000 people die from rabies per year globally, 40% of whom are children under 15. It is endemic primarily in Asian and African countries where most deaths are reported [1–3]. Rabies virus is transferred to humans through bites or scratches by infected domestic and wild dogs and other animals, such as bats, cats, foxes, and many others. Clinical symptoms may include severe encephalitis and hydrophobia. It is transmitted through the saliva of a rabies mammal via skin or mucous membranes and hence causes severe infection [4].

Scratches or minor wounds can potentially be contagious and must be addressed carefully if exposure is possible. Wound disinfection, rabies vaccination, and perhaps rabies immunoglobulin (RIG) therapy should begin as soon as possible to eliminate the virus in the wound before it reaches neurons. Effective postexposure prophylaxis (PEP) in a vaccine provides a strong stimulus, thus inducing neutralizing antibodies to combat the virus. Therefore, it is stated that rabies is unique and different from other infections when clinical illness progression can be averted via prompt care and efficient PEP upon exposure to the rabies virus [5].

Rabies is endemic in Pakistan, caused mainly by dog bites, and diagnosed based on clinical symptoms only. Dog-mediated rabies causes tens of thousands of mortalities annually in the country, which usually remains under-reported. Despite the significant prevalence of dog bites in Pakistan, it is still a neglected disease that is not given enough attention by the health authorities regarding cost-effective treatment options and further research to develop vaccines and antisera with its resources. More than 97,000 dog bite incidents have been documented by the World Health Organization (WHO) in primary care facilities, but most of the cases are not recorded due to a lack of awareness related to rabies complications in the rural population. Unfortunately, there is a lack of data on human rabies cases available at the national level. However, the Health Research Institute (HRI) reported an estimated 2,000 to 5,000 deaths yearly due to rabies in Pakistan [6]. To decrease rabies incidence in Pakistan, an urgent multisectorial approach is required, including training public and healthcare practitioners, especially the awareness to use cost-effective cell culture intradermal (ID) vaccination regimens [7].

Countless deaths resulting from rabies can be avoided by administering timely PEP to individuals bitten by infected dogs. However, the availability of rabies vaccines and purified immunoglobulins remains inadequate in many countries where the disease is prevalent due to exorbitant expenses associated with cell culture vaccines (CCV), human-derived antisera, lack of access, and supply chain issues. Consequently, there is a pressing need for cost-effective, patient-centric, time-efficient, and immunogenic strategies to ensure effective rabies vaccine immunization among humans and animals for active or passive immunity [8].

The high cost of CCVs administered via intramuscular (IM) injection presents a significant obstacle in most developing nations where rabies is endemic. The three WHO prequalified vaccines mostly available in the market include Human Diploid Cell Vaccine (HDCV, IMOVAX™) 1 ml/vial manufactured by (Sanofi), Purified Chick Embryo Cell Vaccine (PCECV) 1 ml/vial (Rabipur/RabAvert, GSK™), and Purified Vero Cell Vaccine (PVRV) 05 ml/vial (Verorab™ by Sanofi) [9].

On the recommendations of the “Strategic Advisory Group of Experts” (SAGE) working group, the WHO has revised its recommendations for PEP and has emphasized adopting the most immunogenic yet cost-effective method of vaccination to achieve the most significant benefit for human vaccination campaigns. Several studies addressed the respective immunogenicity of the ID regimens due to the advantage of using fewer doses and less vaccine consumption compared with previously used IM-based rabies vaccine regimens that require more vaccine and longer duration to achieve the desired RIG titers [10].

WHO suggests that immunologically compromised individuals who have been subjected to category II exposures should receive rabies PEP. It is strongly recommended that the bite site be thoroughly washed, followed by immediate vaccination using one of three regimens: (a) “Institute Pasteur du Cambodia 2-2-2-0-0,” which involves administering two ID sites on days 0, 3, and 7 for a total duration of seven days; (b) “Essen 1-1-1-1-0,” which requires a single IM injection on days 0, 3, and between Day 14 and Day 28 for a maximum duration of up to fourteen to twenty-eight days; or (c) “Zagreb 2-0-1-0-1”, involving two IM injections on Day 0 and one IM injection each on days seven and twenty-one for a total duration of twenty-one days. According to WHO guidelines, category III injuries necessitate using RIG [11, 12].

Thai Red Cross (TRC) updated the regimen (TRC) two-site 28-day (2-2-2-0-2) ID procedure, which was endorsed by the WHO until 2018. This multisite ID regimen was pioneered in Thailand Queen Saovabha Memorial Institute in 1986, popularly known as the TRC regimen [10]; it is a cost-effective alternative and incentive for countries like Pakistan but has a high rabies burden. In addition, Quiambao BP et al. [12] reported another 4-site 1-week ID (ID, 4-4-4-0-0) for PEP using purified Vero cell rabies vaccine (PVRV; Verorab™, Sanofi Pasteur) was noninferior to updated TRC 2-site 28-day (2-2-2-0-2) ID regimen and achieved higher rabies virus neutralization (antibody RVNA) titers [12].

This research aimed to determine the most appropriate vaccination strategy for rabies vaccine to achieve an improved antibody titer with fewer vaccine doses and reduced time. This was achieved by assessing the seroconversion rate of shorter ID vaccination regimens in rabbit models, as opposed to the commonly utilized IM “Essen” regimen for RIG production. It was hypothesized that this approach could be recommended for effective rabies PEP in humans, which may prove beneficial in attaining the required RIG titer in rabbits more quickly and enhancing product yield from animal plasma for local manufacturing of animal-derived immunoglobulins. Such an approach could also be employed on other donor animals for antirabies serum production in countries like Pakistan, where rabies is endemic.

## 2. Materials and Methods

### 2.1. Experimental Animals and Ethical Approval

**Figure 1:**
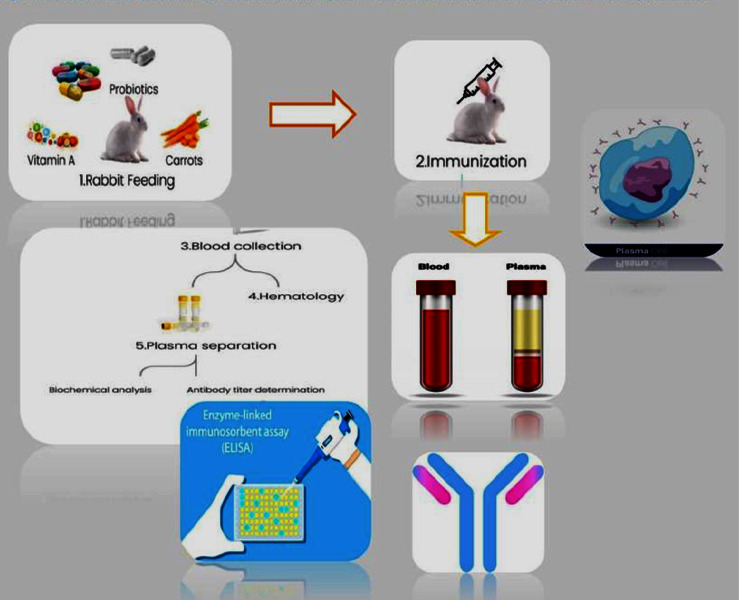
A schematic diagram illustrating the experimental methodology and sequence for immunizing NZW rabbits to produce polyclonal antibodies in response to rabies vaccine, as well as collecting serum from the animals, followed by determining specific antibody titer through utilization of a Platelia II^TM^ ELISA kit designed for rabies.

A total of 24 adult NZW rabbits were taken from the National Institute of Health (NIH) Animal House Division. Six animals were included in each of the four groups (*N* = 24). Rabbits were reared in well-ventilated animal houses under suitable conditions, and a standard diet with added vitamin A and probiotics supplementation was given to them. The NIH Ethics Committee approved the study protocol, Islamabad (Ethical Approval No. F.1-5/ERC/2020). The research animals were immunized with a CCRV obtained from the NIH. This investigation excluded animals exhibiting any indications of sluggishness. As shown in Figure 1, animal feeding, immunization, blood sampling, and serological testing were carried out according to the standard protocols.

### 2.2. Experimental Design

A randomized and controlled study was carried out on male NZW rabbits. Rabbits aged between 5 and 6 weeks old, weighing 1.5 to 2 kg were chosen for the experiment based on their superior antibody response capability. All rabbits were raised in a conventional animal care facility at NIH, adhering to standard operating procedures (SOPs).  Table 1 outlines the immunization protocol for each of the four groups of NZW rabbits (*n* = 24 additional rabbits were included for immunization). The first group served as a control (R1—control group) and received vaccinations using “Essen regimen” intramuscularly with doses administered as follows: 1-1-1-1-1. The second group (R2) was vaccinated using the two-site Institute Pasteur du Cambodia ID regimen with doses administered as follows: 2-2-2-0-0. For the third group (R3), we selected a four-site one-week alternate ID regimen with doses administered as follows: 4-4-4-0-0, while the fourth group was immunized with an updated TRC-ID regimen and received dosages of 2-2-20-0-2, which is shown in Table 1 [12]. Blood samples were collected at regular intervals, namely, days zero, fourteen, and thirty-five, for detailed analysis, including hematology, biochemical parameters, and seroconversion studies to determine rabies antibody levels, among other factors.

### 2.3. Vaccine for Immunization of Animals

The CCRV Vero-cell-based rabies vaccine was produced by the Biological Production Division (BPD) of NIH Pakistan. Each dose contained a pure and nonactive strain of the rabies virus with >2.5 IU/dose, which meets WHO standards for human consumption. The control R1 group received the CCRV via IM injection at the recommended dosage, whereas experimental groups were immunized using insulin syringes and needles at a 0.1 ml dose per injection site for ID administration.

### 2.4. Animal's Immunization

**Figure 2:**
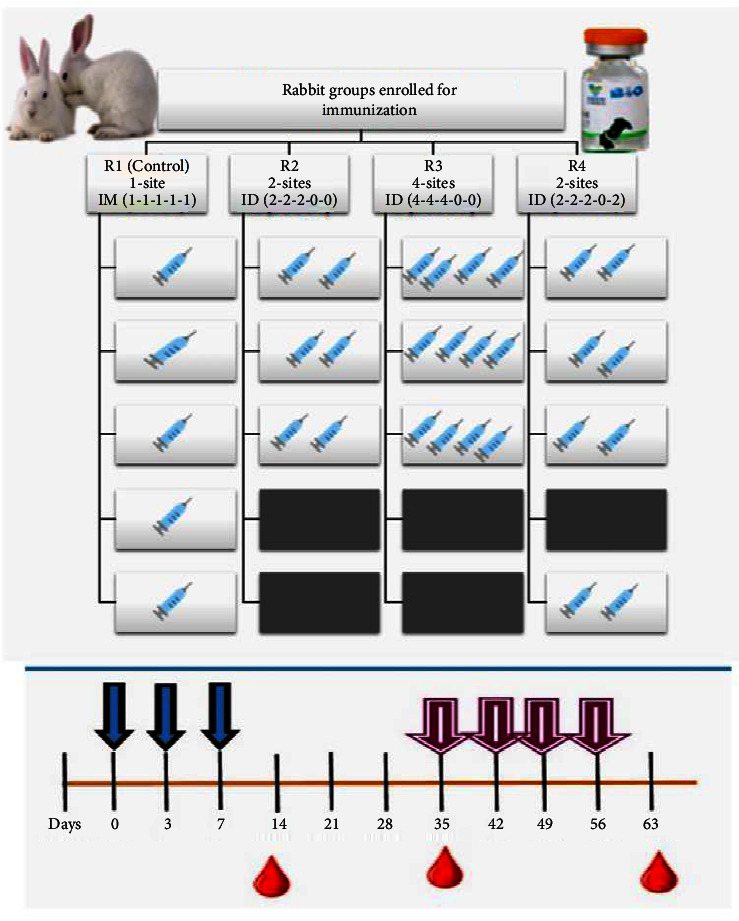
Schedule displaying immunization and blood sampling scheme of animals from each group. All animals (*n* = 24) were initially immunized on days 0, 3, and 7 and afterward as per the recommended schedule. For hyperimmunization, booster doses were administered weekly after the scheduled dosing on days 35, 42, 49, and 56. Blood sample collection was done on days 14 and 35, while final bleeding was carried out on Day 63 for enriched plasma collection.

All animals were immunized at deltoids and interior thighs on specific days. Three regimens based on the 2-site ID TRC postexposure regimens recommended by [11] were selected per the following grouping (Table 1 and Figure 2).

The NZW rabbits (*n* = 24) were randomly divided into R1 (control group) and three experimental groups (R2, R3, and R4) as per immunization strategy and number of injection sites. (a) R2: 2-site 1-week (IPC (Institut Pasteur du Cambodge)) ID regimen, (b) R3: 2-site 28-day TRC-ID regimen, and (c) R4: 4-site one-week regimen (alternate ID regimen), respectively, and compared with five doses of standard Essen IM schedule of rabies vaccine at days 0, 3, 7, 14, and 28 in the control group R1. Results were evaluated at days 14 and 35 postvaccination. Mean antibody titer was determined using the rabies-specific Platelia II™ ELISA kit method by sampling at days 14 and 35. All animals were fed a vitamin A-rich diet containing organic carrots and commercial probiotic supplements in daily feed.

### 2.5. Immunization of R1 Control Group (Essen IM Regimen)

The primary IM postexposure vaccine regimen recommended by WHO, the 5-dose Essen (days 0, 3, 7, 14, and 28), was selected as the control in the R1 group. As shown in Figure 1, five doses of 1 ml were injected intramuscularly at regular intervals (1-1-1-1-1) at each site for 28 days. A total vaccine volume of 0.5 ml dose per site was injected.

### 2.6. Immunization of the R2 Group

The 2-site ID 1-week IPC-ID regimen, including 1 ml at 2 ID sites on days 0, 3, and 7, was used for immunization as per standard recommendations.

### 2.7. Immunization of R3 Group (Alternate/4-Site One-Week ID Regimen)

The alternative recommendations include a 4-site ID 1-week regimen, double the dose of the 2-site principle TRC recommendation: 1 ml ID injections at 4 sites on days 0, 3, and 7 were done.

### 2.8. Immunization Scheme for Group R4 (TRC-ID Regimen)

The principle TRC-ID regimen was used to immunize rabbits in group R4 with 2-site injections for approximately 1 month, with a missing dose on Day 21 (Table 1 and Figure 1).

### 2.9. Hyperimmunization Schedule for RIG Production

For the hyperimmunization of animals, the method described by Queen Saovabha Memorial Institute, Bangkok, Thailand, was applied with modifications [10]. After 28 days of vaccination, selected animals were immunized weekly as a booster dose at days 35, 42, 49, and 56 with two human doses of CCRV. From Day 21 to Day 56, serum samples were taken weekly, and the ELISA test method investigated the antibody titer. Animals showing a lower titer than 70 lU/ml by Day 56 were withdrawn from the donor animal population. On Day 63, the bleeding of animals was carried out for further plasma purification for collecting RIGs [13].

### 2.10. Blood Sampling and Serum Separation

Totally, 2-3 ml of whole blood sampling from an ear marginal vein of rabbits was done on Day 14 in all groups and one week after the last dose on Day 28. Sera were separated by centrifugation, and samples were preserved at +2 to 8°C for further antibody detection tests.

### 2.11. Determination of Physical and Clinical Parameters and Blood Sampling for Test Analysis

Approximately 2 ml venous blood samples were obtained on days 0, 14, and 35 after complete vaccination for serology testing (hematology, biochemistry, and antibody titer). Mortality, physical fitness observation, and weight calculation for animal general health were monitored at weekly intervals from Day 0 till experiment completion. Biochemical testing was performed on the Cobas C311 machine using Roche standardized kits according to the manufacturer's instructions. Moreover, local and systemic reactions were monitored after each immunization day to observe any adverse reactions.

### 2.12. Rabies Serology for Rabies Antibody Titer Determination

Blood samples were collected in EDTA tubes for maximum plasma antibody titer calculation using the Platelia II™ ELISA kit purchased from Bio-Rad. Protocol for detecting antibodies by the ELISA kit method was followed according to the manufacturer's instructions. The buffer and solutions were prepared using analytical reagent-grade chemicals unless specified.

### 2.13. Antibody Screening Procedure Using the ELISA Kit Technique

The Platelia II™ kit was used in an indirect immune-enzymatic assay to identify rabies virus antiglycoprotein antibodies in rabbit serum samples. The detection was carried out following the manufacturer's instructions. This test was chosen due to its quick turnaround time (three hours), easy use, and qualitative and quantitative findings supply. Moreover, the test had 99% sensitivity and 99.4% specificity [14].

The serum samples were reduced in concentration by dissolving 10 *µ*l of samples in 990 *µ*l of diluting solution. The screening procedure entailed distributing controls (negative and positive), serum samples, and measurement standards into microplates and incubating them for an hour at 37°C. Three washing procedures were carried out after incubation to ensure the elimination of unattached antibodies and additional proteins from the samples, and 100 *µ*l of conjugate-protein A labeled with peroxidase was added to each well. The microplate was incubated for one hour at 37°C before being washed five times to eliminate the free conjugate. The plate was kept in an incubator at 25°C for 30 minutes with the addition of per-oxidase substrate and chromogen before including a 100 *µ*l solution of 1N H_2_SO_4_. Then, optical density (OD) was obtained at 450 and 620 nm using a microplate reader (Diateck China).

**Figure 3:**
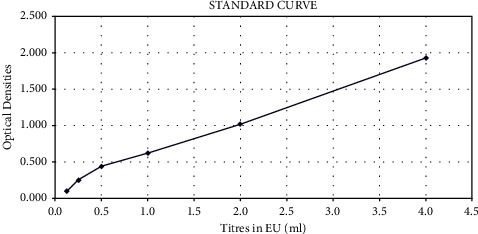
Standard curve for calculating the quantitative titer of antirabies antibodies. By diluting measurement standards, the standard curve was developed. The serum titer values were determined by comparing the OD values for the unknown sample with those of the positive controls. The quantified sera titer was indicated by the equivalent units per milliliter (EU/ml), and a direct reading on the standard curve correlated to this value. Titer values were determined using the given conversion software tool. At titer values of 4, 0.5–4, 0.125–0.5, and 0.125 EU/ml, the results were classified as high seroconversion, sufficient seroconversion, insufficient seroconversion, and undetectable seroconversion.

The rabies antibodies were quantitatively determined by constructing a standard curve for quantification standards as shown in Figure 3 (S1–S6: prepared by serially diluting R4b-calibrated positive controls), and results were categorized based on seroconversion level at days 14 and 35 postvaccination as shown in Table 2.

The OD values for the unknown sample were contrasted to positive controls, which supported the calculation of sera titer. A precise reading on the standard curve represented the measured sera titer as equivalent units per ml (EU/ml). The results were categorized based on high seroconversion level, sufficient seroconversion, insufficient seroconversion level, and undetectable seroconversion having greater than 4, 0.5–4, 0.125–0.5, and below 0.125 EU/ml titer, respectively.

### 2.14. Statistical Analysis

All data were analyzed using the GraphPad Prism 9 software (GraphPad Software, Inc., La Jolla, CA, USA). All data are presented as a mean ± standard deviation (SD) and were first checked for normality using the Kolmogorov–Smirnov and the D'Agostino–Pearson tests. A one-way ANOVA followed by the Tukey multiple comparison test was used to compare differences among antibody titer values of each study animal at 14 days and 35 days. A two-way ANOVA followed by Tukey's multiple comparison test was used to compare mean serum OD values and rabies-neutralizing antibody titer at days 14 and 35 among groups immunized with different rabies vaccine regimens. The level of significance has been set at *p* < 0.05.

## 3. Results and Discussion

The study aimed to assess the seroconversion against rabies vaccine using shorter intradermal vaccination regimens consisting of 3 to 4 doses. These regimens were based on the two-site ID TRC and were compared with the commonly used “Essen” regimen, which consists of a five-dose IM injection. The primary aim was to achieve rabies antibody titer levels well above the recommended threshold value, i.e., 0.5 EU/ml, followed by enhancing these values using a more immunogenic ID regimen for polyclonal RIG production in NZW rabbit models. Seroconversion levels were evaluated through an ELISA kit method that measured OD and calculated antibody titer values in EU/ml using a titer conversion tool. Results from groups R2, R3, and R4 were compared with those from control group R1 at days 14 and 35 postvaccination.

The immunological response in rabbits against the rabies vaccine administered from IM and ID was determined. The mean ODs obtained by the ELISA method with PLATELIA II kit for R1, R2, R3, and R4 were 1.51, 1.69, 1.79, and 1.64 OD, respectively, after 14 days, as shown in Table 3.

**Figure 4:**
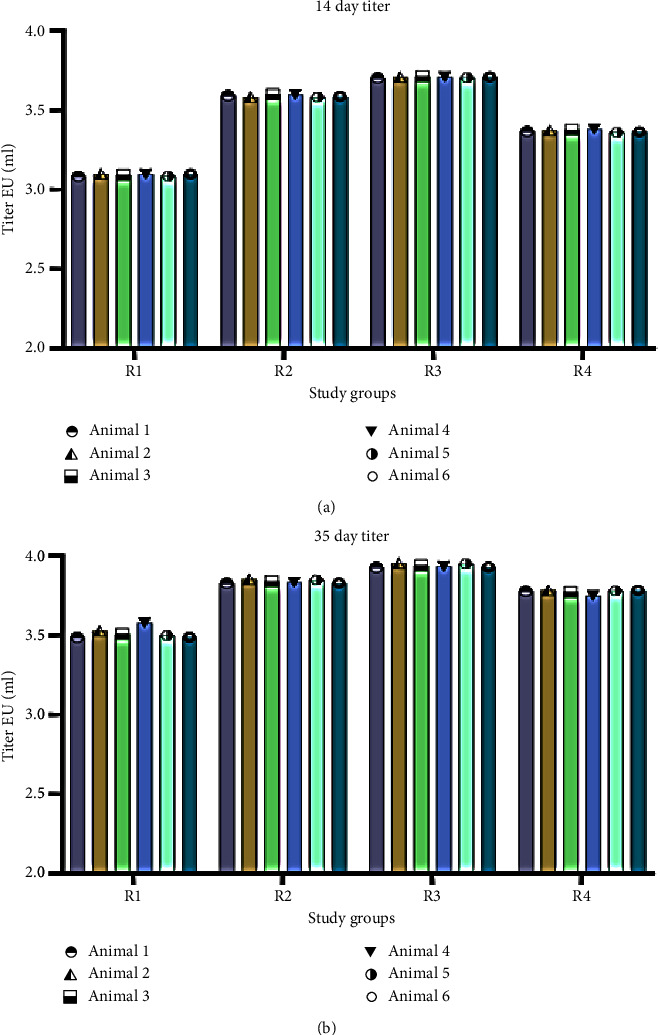
Antibody titer values of each study animal, (a) at 14 days and (b) at 35 days.

This shows an increase of 8.06-18.16% in OD, representing immunologic response between the group R1 with R2, R3, and R4, indicating a higher seroconversion and immune response from ID dose in groups R2 and R3 compared to R1 (control), even after one week of immunization (*p* < 0.001). Moreover, the increase in OD between groups R1 and R2 presents an 11.48% increase in immune response, indicating a significant titer from ID compared to IM (*p* < 0.001). The OD values for R1, R2, R3, and R4 after 35 days of immunization were 1.69, 1.80, 1.85, and 1.82 OD. The results show a significantly higher OD value for ID than IM (*p* < 0.05). In addition, a comparatively high difference in titer was obtained between the control and R2 (*p* < 0.001). Similarly, the OD increase in the R1 group from 14 to 35 days was 11.43%, whereas the OD increase in groups R2, R3, and R4 was in the range of 19–22%, presenting a highly substantial response of ID vaccination in comparison with IM (*p* < 0.01) (Table 3 and Figure 4). Seroconversion rate is the proportion of vaccines achieving rabies virus-neutralizing antibody (RVNA) titers ≥0.5 IU/ml.

**Figure 5:**
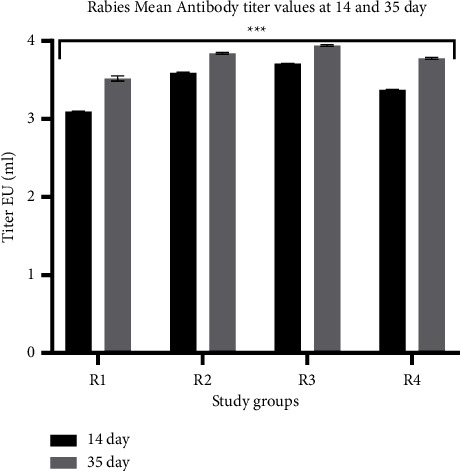
Mean rabies-neutralizing antibody titer at days 14 and 35 among groups immunized with different rabies vaccine regimens. Antibody titer was represented as EU/ml as determined by the ELISA kit conversion tool (^*∗∗∗*^*p* < 0.0001).

Immune response in terms of antibody titer was calculated from OD values with the conversion tool provided with the kit. For the four groups (R1, R2, R3, and R4), antibody titer values were 3.094, 3.591, 3.709, and 3.371 EU/ml, respectively, after 14 days (Figure 5). All groups showed seroconversion above the recommended threshold of protective titer values on Day 14 (Table 2). R3 showed the highest titer compared to other groups. Similarly, experimental groups immunized through ID doses showed higher antibody titer than the R1 control group. All the groups reached “protective” rabies virus-neutralizing antibody titer >0.5 IU/ml on Day 14. After 35 days, antibody titers for R1, R2, R3, and R4 were 3.516, 3.841, 3.940, and 3.776 IU/ml, respectively. The antibody titers indicate a significant increase in regimen immunized intradermally (R2, R3, and R4), all having titers above 3 IU/ml. However, groups immunized within one week (R2 and R3) presented the highest titer of >3.6 IU/ml on Day 14, whereas R3 showed a 3.371 IU/ml titer. Moreover, on Day 35, antibody titers among all the groups reached >3.5 IU/ml. A similar trend of increased antibody titer among the three groups (R2, R3, and R4) was observed even after 35 days, suggesting ID routes were noninferior over IM (Table 4). Although the titer on Day 35 was more than that observed on Day 14, high seroconversion was attained since Day 14. This could also be due to the provision of dietary supplements and probiotics.

The results suggest a significant immune response in less course of time among rabbit groups immunized intradermally (ID at multisites) than control group R1 (5-dose, single-site IM immunization for one month). Among all animals, rabbits of R2 and R3 show significant antibody titers after a one-week immunization course. However, results shown by R2 (IPC 2-site 1-week regimen was crucial as it gives antibody titer of 3.59 IU/ml after 14 days upon consumption of the least dose experimented), i.e., 0.6 ml. Using less dosage would make it a more cost-effective method for better yielding the final product (RIG) from donor rabbits.

WHO recommendations for using ID administration of rabies vaccine for PEP were based on many reported advantages. However, the cost and availability of rabies vaccine limit the use of prophylactic rabies vaccination in developing countries, hence urging to explore alternative vaccination strategies. The seroconversion rates on Day 14 in groups immunized with ID were noninferior to IM. A similar response was found after 35 days, suggesting that ID immunization was noninferior. Moreover, all the rabbits were given high vitamin-rich probiotic dietary supplementation, contributing to the improved humoral response [15–20]. It was reported that ID immunization, even with a low dose, delivers antigen directly to the area that contains a high number of antigen-presenting cells (APCs), thus producing higher antibody titers than the previously used IM vaccination regimen. This contributes to the cost-effectiveness of the ID vaccination strategy as fewer vaccine doses could produce a noticeable antibody response in the plasma of the donor animal [21].

Our experimental study concluded that a shorter ID (2-site 1-week) regimen was noninferior and more advantageous over an extensively used IM schedule. Due to its high seroconversion response within 14 days, as well as the application of a minimum dose compared to IM, ID provides an equally safe and immunogenic substitute for IM vaccination. Only a few vaccine vials are needed to complete a full course of effective immunization; hence, hyperimmunization provides a better alternative for RIG production.

## 4. Conclusions

The expense of cell culture rabies vaccines (CCRVs) for intramuscular (IM) administration restricts their use in resource-limited countries due to delayed antibody response postimmunization and higher vaccine volume consumption. The International Health Organization (WHO)-endorsed intradermal (ID) schedule of RVs offers several benefits, including cost-effectiveness and better patient compliance with fewer clinical visits. Hence, global health authorities encourage high-burden adoption and implementation of ID schedules by national authorities. Generally, the ID route was considered more immunogenic and demonstrated safety, efficacy, and cost-effectiveness. Therefore, it was preferable for active human or animal immunization and passive immunization through rabies immune globulin (RIG) production plans. Furthermore, multisite ID vaccination programs can effectively enhance RIG production in animals to address the shortage of RIGs in highly affected developing nations.

## Figures and Tables

**Table 1:** Distribution of the tested animals among distinct groups according to vaccination schedules.

Group ID	Vaccination regimen applied	Immunization days 0-3-7-14-28	Sites of injection	Total vaccine vials used/animal
R1 (control)	Essen IM single dose 0.5 ml	1-1-1-1-1 28 days	1-site	5 vials

R2	IPC (ID) 0.1 ml/site	2-2-2-0-0 7 days	2-site	1.1 vials

R3	TRC (ID) 0.1 ml/site	2-2-2-0-2 28 days	2-site	1.1 vials

R4	Alternate (ID) 0.1 ml/site	4-4-4-0-0 7 days	4-site	3 vials

Booster doses: as recommended by WHO for polyclonal RIG production.

**Table 2:** Level of seroconversion in all study groups at days 14 and 35.

	R1	R2	R3	R4
DAY 14	Seroconverted	Improved/sufficient seroconversion	High seroconversion	High seroconversion
DAY 35	Sufficient seroconversion	High seroconversion	High seroconversion	High seroconversion

*Note.* SSC: sufficient seroconversion; HSC: high seroconversion.

**Table 3:** Comparison of mean serum OD values obtained by ELISA from study groups at 14 and 35 days.

Day	Regimens	Mean OD	Variance	*t*-value	*p* value (two-tailed)
14	Control	1.5046	3.63*E* − 05	4.3027	0.0006
	R2	1.6983	0.0001		
	Control	1.5046	3.63*E* − 05	4.3027	0.0002
	R3	1.7813	0.0001		
	Control	1.5046	3.63*E* − 05	4.3027	0.0027
	R4	1.6376	3.63*E* − 05		

35	Control	1.687	0.0001	4.3027	0.0016
	R2	1.7976	6.33*E* − 06		
	Control	1.687	0.0001	4.3027	0.0031
	R3	1.839	3.6*E* − 05		
	Control	1.687	0.0001	4.3027	0.0314
	R4	1.802	0.0007		

**Table 4:** Summary table on the effect of days on rabies mean antibody titer values.

	Source of variation	SS	df	MS	*F*	*p* value	*F* crit
Antibody titer	Between groups	0.09513	1	0.09513	12.81386	0.001671	4.30095
	Within groups	0.163328	22	0.007424			
	Total	0.258458	23				

## Data Availability

The data used to support the findings of this study are available from the corresponding author upon request.

## References

[1] Hampson K., Coudeville L., Lembo T. (2015). Estimating the global burden of endemic canine rabies. *PLoS Neglected Tropical Diseases*.

[2] Taylor L. H., Hampson K., Fahrion A., Abela-Ridder B., Nel L. H. (2017). Difficulties in estimating the human burden of canine rabies. *Acta Tropica*.

[3] Who (2005). Expert consultation on rabies. *World Health Organization Technical Report Series*.

[4] Soler-Rangel S., Jimenez-Restrepo N., Narino D., Rosselli D. (2020). Rabies encephalitis and extra-neural manifestations in a patient bitten by a domestic cat. *Revista do Instituto de Medicina Tropical de São Paulo*.

[5] Rupprecht C. E., Salahuddin N. (2019). Current status of human rabies prevention: remaining barriers to global biologics accessibility and disease elimination. *Expert Review of Vaccines*.

[6] Sulehri M. A., Riaz O., Hussain R. (2011). Rabies: a highly fatal but preventable disease. *A.P.M.C.*.

[7] Parviz S., Chotani R., McCormick J., Fisher-Hoch S., Luby S. (2004). Rabies deaths in Pakistan: results of ineffective post-exposure treatment. *International Journal of Infectious Diseases*.

[8] Sreenivasan N., Li A., Shiferaw M. (2019). Overview of rabies post-exposure prophylaxis access, procurement and distribution in selected countries in Asia and Africa, 2017-2018. *Vaccine*.

[9] Warrell M. J. (2019). Rabies post-exposure vaccination in 2 visits within a week: a 4-site intradermal regimen. *Vaccine*.

[10] Gongal G., Sampath G. (2019). Introduction of intradermal rabies vaccination-A paradigm shift in improving post-exposure prophylaxis in Asia. *Vaccine*.

[11] Who (2018). Rabies vaccines: WHO position paper, April 2018-Recommendations. *Vaccine*.

[12] Quiambao B. P., Ambas C., Diego S. (2020). Single-visit, 4-site intradermal (ID) rabies vaccination induces robust immune responses 5 years after 1-week, 4-site ID primary post-exposure prophylaxis in the Philippines. *Vaccine*.

[13] Rupprecht C. E., Xiang Z., Servat A., Franka R., Kirby J., Ertl H. C. J. (2018). Additional progress in the development and application of a direct, rapid immunohistochemical test for rabies diagnosis. *Veterinary Sciences*.

[14] Feyssaguet M., Dacheux L., Audry L. (2007). Multicenter comparative study of a new ELISA, PLATELIA RABIES II, for the detection and titration of anti-rabies glycoprotein antibodies and comparison with the rapid fluorescent focus inhibition test (RFFIT) on human samples from vaccinated and non-vaccinated people. *Vaccine*.

[15] Di Cerbo A., Palmieri B. (2015). Review: the market of probiotics. *Pakistan journal of pharmaceutical sciences*.

[16] Najam A., Ahmad S., Abid R. (2023). Immune-adjuvant effect of vitamin A and probiotics supplementation on humoral response to cell culture rabies vaccine in rabbits. *3 Biotech*.

[17] Abid R., Waseem H., Ali J. (2022). Probiotic yeast Saccharomyces: back to nature to improve human health. *Journal of Fungi*.

[18] Abid S., Farid A., Abid R. (2022). Identification, biochemical characterization, and safety attributes of locally isolated lactobacillus fermentum from Bubalus bubalis (buffalo) milk as a probiotic. *Microorganisms*.

[19] Idrees M., Imran M., Atiq N. (2022). Probiotics, their action modality and the use of multi-omics in metamorphosis of commensal microbiota into target-based probiotics. *Frontiers in Nutrition*.

[20] Di Cerbo A., Palmieri B., Aponte M., Morales-Medina J. C., Iannitti T. (2016). Mechanisms and therapeutic effectiveness of lactobacilli. *Journal of Clinical Pathology*.

[21] Wangmo K., Laven R., Cliquet F., Wasniewski M., Yang A. (2019). Comparison of antibody titres between intradermal and intramuscular rabies vaccination using inactivated vaccine in cattle in Bhutan. *PLoS One*.

